# Outcomes following use of VersaWrap nerve protector in treatment of patients with recurrent compressive neuropathies

**DOI:** 10.3389/fsurg.2023.1123375

**Published:** 2023-03-21

**Authors:** Keegan M. Hones, David Spencer Nichols, Haley Barker, Elizabeth Cox, Jaime A. Hones, Harvey Chim

**Affiliations:** ^1^ University of Florida Collage of Medicine, Gainesville, FL, United States; ^2^ Department of Plastic and Reconstructive Surgery, University of Florida, Gainesville, FL, United States; ^3^ Department of Neurosurgery, University of Florida, Gainesville, FL, United States

**Keywords:** nerve wrap, nerve protector, peripheral nerve, carpal tunnel, cubital tunnel, radial tunnel

## Abstract

Epineural scarring following previous carpal or cubital tunnel release can lead to pain and permanent dysfunction. To prevent this cascade, nerve wraps are an option. The purpose of this study was to evaluate outcomes following use of VersaWrap nerve protector during surgical decompression and neurolysis in patients with recurrent compressive neuropathies in the upper extremity. Twenty patients comprised the patient cohort, with a mean postoperative follow-up time of 139 days (range: 42–356 days). There were 13 females and 7 males, with a mean age of 43.4 years. Fourteen surgeries were performed for revision cubital tunnel, 5 for revision carpal tunnel, and 1 for revision radial tunnel syndrome. Average duration of symptoms prior to revision surgery with VersaWrap was 2 years (range 9 months to 6 years). Postoperatively, the mean DASH score was 57.7 and VAS 3.1. Mean s2PD median distribution was 7.3, s2PD ulnar distribution 8.9, m2PD median distribution 6.9 and m2PD ulnar distribution 7.3. All patients had subjective improvement of symptoms and were satisfied with their result. No patients in our cohort required further revisional surgery. In conclusion, the use of VersaWrap as a nerve protector following revision surgery for recurrent compressive neuropathies in the upper extremity was safe and effective.

**Level of Evidence:** IV; retrospective case series

## Introduction

Epineural scarring following previous carpal or cubital tunnel release can result in symptomatic recurrence, with pain, numbness, and weakness. Recurrent formation of scar tissue around a peripheral nerve is a particular issue in revision surgery. To reduce scarring around the nerve, many surgeons use nerve wraps or conduits. The efficacy of nerve protectors for treatment of different nerve pathologies has been demonstrated in both animal and human models (1–7). Absorbable nerve wraps are favored, with the benefit conferred by these implants likely related to the separation of soft tissue and nerve, allowing gliding and thus preventing tethering, and providing an optimized environment for nerve healing. However, disadvantages of some commercially available wraps or conduits include unfavorable handling characteristics, stiffness, and added bulk which can result in nerve constriction. In some cases where a nerve wrap is sutured in place around a peripheral nerve, postoperative swelling can result in a localized constriction point which results in persistent neuropathic pain and resultant disability.

VersaWrap (Alafair Biosciences, Austin, Texas, USA) is a bioresorbable plant-based hydrogel wrap that was recently approved in the USA for use as a nerve wrap. Originally described for use as a wrap following repair of tendon injuries, VersaWrap was subsequently also used for treatment of peripheral nerve injuries, particularly in cases where scarring was a concern. VersaWrap consists of an ultrathin hydrogel sheet composed of hyaluronic acid and alginate, which provides a pliable wrap and non-constricting interface for tendon and peripheral nerves (8). Some advantages of VersaWrap favoring its use as a nerve wrap include its ultrathin conformable nature, smooth hydrophilic surface facilitating nerve gliding, ability to be placed without sutures, lack of polarity and transparent appearance which allows visualization of the underlying wrapped nerve. In addition, Versawrap can be tailored to fit any diameter peripheral nerve. VersaWrap is bioresorbed *via* hydrolysis and metabolic activity, unlike collagen-based implants that are remodeled and thus result in added bulk over time at the nerve repair site. Finally, as VersaWrap has no animal-derived or human tissue component, there is decreased concern for immune response and disease transmission.

Here we describe the first published experience, to our knowledge, utilising VersaWrap as a peripheral nerve wrap. In our series, VersaWrap was used primarily as a nerve protector in recurrent compressive neuropathies in the upper extremity, to reduce postoperative scarring. This remains of particular relevance given that reported rates of symptom recurrence after primary carpal tunnel surgery range from 1 to 31% (6, 9, 10) and 2.4% to 25% after primary cubital tunnel surgery (11, 12). Revision peripheral nerve surgeries involve significant scar tissue, and present significant, unique challenges. In this study, we retrospectively reviewed a series of patients in whom VersaWrap was utilized for revision upper extremity peripheral nerve decompression and neurolysis, with a focus on analysis of clinical outcomes.

## Materials and methods

**Figure 1:**
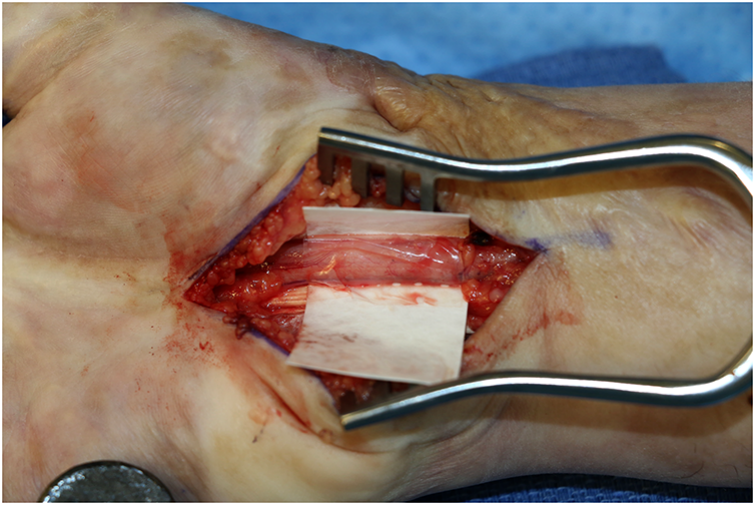
Versawrap has been placed around the median nerve following revision carpal tunnel release. The pliable and transparent nature of VersaWrap facilitates placement as a circumferential nerve wrap.

**Figure 2:**
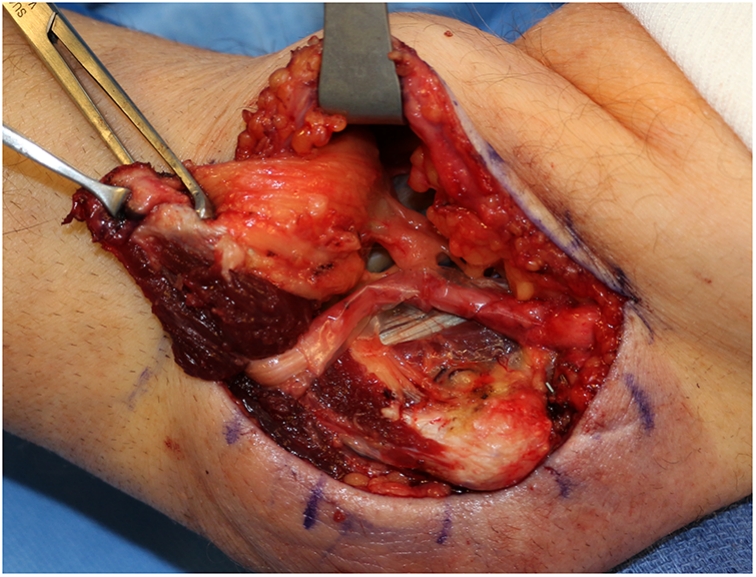
Versawrap has been placed around the ulnar nerve following revision cubital tunnel release, in preparation for submuscular transposition. The pliable and transparent nature of VersaWrap facilitates placement as a circumferential nerve wrap.

A single institution retrospective review was performed to identify all patients who received VersaWrap as a nerve protector intraoperatively for recurrent compressive neuropathies of the upper extremity. The study was approved by our institutional review board with a unique identification number of IRB202001859. All procedures were in accordance with the ethical standards of the responsible committee on human experimentation, the Helsinki Declaration of 1964, and later versions. Informed consent was obtained from all patients for their participation in the study. From February 2020 to June 2022, a total of 41 patients had VersaWrap used following surgical treatment of recurrent carpal tunnel syndrome, cubital tunnel syndrome or other unusual compressive neuropathies. Intraoperative use of VersaWrap nerve protector is illustrated through representative examples in Figures 1, 2. Twenty patients agreed to return for in-person postoperative assessment, and this comprised the patient cohort for this study. All patients had at least 6 weeks postoperative follow-up.

All surgeries were performed by the senior author. Demographic data collected included age at surgery, gender, body mass index (BMI), history of Diabetes Mellitus and smoking status. Outcome measures included static and moving two-point discrimination (s2PD and m2PD), range of motion (ROM), and power of affected muscles as assessed using the Medical Research Council (MRC) grading scale. Standardized outcome scores including the Disabilities of the Arm, Shoulder, and Hand (DASH) questionnaire and Visual Analog Scale (VAS) were assessed postoperatively. The DASH score was chosen as this is a commonly used patient reported outcome measure in hand surgery.

## Results

We evaluated 20 patients treated for upper extremity peripheral nerve injuries who had VersaWrap utilized intraoperatively. The average age of patients in our series was 43.4 years (range: 21–69). There were 13 females and 7 males. Mean follow-up time was 139 days (range: 42–356 days). All had previous surgery to the surgical site. Average duration of symptoms prior to revision surgery with VersaWrap placement was 2 years (range 9 months to 6 years). Of the 20 included, 14 surgeries were for revision cubital tunnel, 5 for revision carpal tunnel, and 1 for revision radial tunnel syndrome. Three patients were smokers, 3 former smokers, and 14 non-smokers. One patient in the cohort had diabetes mellitus. The dominant extremity was the affected side in 14/20. Mean BMI was 26.6. There were no intraoperative complications in the cohort. At the time of follow-up assessment, all patients were satisfied with the outcome and there were no further revision surgeries required.

The mean DASH score for the revision cubital tunnel group was 54.0 and mean VAS 2.7. For the revision carpal tunnel group, the mean DASH was 66.2 and mean VAS 4.2. For the revision radial tunnel patient DASH was 68.3 and VAS 3. The total mean DASH score for the cohort was 57.7 and total mean VAS was 3.1. For the entire cohort, mean s2PD in the median nerve distribution was 7.3, mean s2PD in the ulnar nerve distribution was 8.9. Mean m2PD in the median nerve distribution was 6.9, mean m2PD in the ulnar nerve distribution was 7.3.

In the revision cubital tunnel group, 14/14 patients achieved 75 degrees of wrist flexion, 13/14 achieved 70 degrees of wrist extension, 12/14 achieved full finger abduction and adduction, and 13/14 achieved full interphalangeal joint motion. Mean MRC grade for thumb opposition was 4.8, finger adduction 4.3, finger flexion 4.7, and wrist flexion 4.9. In the revision carpal tunnel group, all patients achieved 75 degrees of wrist flexion, 4/5 achieved 70 degrees of wrist extension, 5/5 achieved full finger abduction and adduction, and 3/5 achieved full interphalangeal joint motion of digits 1–5. Mean MRC grade for thumb opposition was 4.2, finger adduction 4.6, finger flexion 4.2, and wrist flexion 4.8.

For the radial tunnel patient, range of motion achieved was 75 degrees of wrist flexion, 70 degrees of wrist extension, full finger abduction and adduction, but full interphalangeal joint motion was not achieved. MRC grading for thumb opposition was 5, finger adduction 5, finger flexion 5, and wrist flexion 5. In addition, this patient presented preoperatively with weakness of finger extensors, MRC grade 2. Postoperatively, finger extension strength improved to MRC grade 3. These results are summarized in Table 1.

**Table 1:** Outcome measures in patient cohort.

	Achieved full wrist flexion	Achieved full wrist extension	Achieved full finger abduction and adduction	Achieved full interphalangeal joint motion of digits 1–5	Mean thumb opposition (MRC grade)	Mean finger adduction (MRC grade)	Mean finger flexion (MRC grade)	Mean wrist flexion (MRC grade)	DASH	VAS
Cubital tunnel	14/14	13/14	12/14	13/14	4.8	4.3	4.7	4.9	54.0	2.7
Carpal tunnel	5/5	4/5	5/5	3/5	4.2	4.6	4.2	4.8	66.2	4.2
Radial tunnel	1/1	1/1	1/1	0/1	5	5	5	5	68.3	3
Total	20/20	18/20	18/20	16/20	4.7	4.5	4.5	4.9	57.7	3.1

MRC, medical research council; DASH, disabilities of the arm, shoulder, and hand; VAS, visual analog score.

## Discussion

Compressive neuropathies of the upper extremity are very common, with over 400,000 carpal tunnel releases (13, 14) and over 15,000 cubital tunnel releases (15) performed annually. Despite overall good results, some patients continue to have pain, altered sensation or weakness after surgery or experience delayed recurrence of symptoms. Revision surgery may become necessary to alleviate these symptoms, but these surgeries are not generally as straightforward as the initial ones. Scar tissue and altered anatomy present unique challenges for the surgeon. Additionally, epineural scarring is likely more of a risk following a revision surgery. Nerve wraps or conduits may provide specific benefit in these instances. In this paper we present the first published experience, to our knowledge, describing the use of VersaWrap as a nerve protector. In our series, VersaWrap was used for patients with recurrent compressive neuropathies in the upper extremity requiring surgical intervention.

The use of nerve wraps in peripheral nerve surgery is well-established (2, 4, 5). However, as one animal study by Nicolas et al. demonstrated, the potential for over-tightening nerve wraps has detrimental effects (16). Spielman et al. reported resolution of symptoms and improved VAS scores in 30 patients with recurrent or persistent carpal tunnel syndrome who had surgery that involved the use of a nerve wrap (17). Additionally, Thakkar et al. in a systematic review reported improvements in outcomes after revision compressive nerve surgery that included the use of a nerve wrap (18). Other techniques for preventing scarring or recurrence in revision surgery may include the use of local flaps for revision carpal tunnel release of subcutaneous or submuscular transposition for revision cubital tunnel release.

Overall, while this study provides new insights, it does have limitations. This was a retrospectively collected series, with shortcomings inherent to its design. These include a smaller sample size and a single institution experience which may limit generalizability of findings. In addition, as only 20 patients agreed to return postoperatively for data collection, this introduces a source of bias. Finally, we were not able to obtain preoperative DASH and VAS scores due to resource limitations in our clinic. Nevertheless, our findings provide evidence for use of VersaWrap as a safe and effective nerve protector for revision surgery targeted at compressive neuropathies. Hopefully, this data will provide a basis for larger studies and randomized controlled trials.

In conclusion, VersaWrap is safe and effective when used as a nerve wrap. In this study, we focused on patients undergoing revision surgery for compressive neuropathies of the upper extremity. Notably, all patients in this cohort had subjective and objective improvement of symptoms, postoperative satisfaction with results and did not require revisional surgery in the post-operative period examined during this investigation.

## Data Availability

The raw data supporting the conclusions of this article will be made available by the authors, without undue reservation.
